# Supplementary material for: Characteristics and outcomes of pregnant women with placenta accreta spectrum in Italy: A prospective population-based cohort study
Source: PLoS One. 2021 Jun 4;16(6):e0252654. doi: 10.1371/journal.pone.0252654 (PMC8177430; doi:10.1371/journal.pone.0252654)
Supplement: Attachment — Submitted filename: Response to Reviewers.docx [file pone.0252654.s001.docx]

Manuscript requests from the Academic Editor Dr. Clive J Petry, PhD.

We have included a revised statement regarding data availability.

Precisely, data cannot be shared publicly because of ethical standards and legal requirements. Data are available from the Ethics Committee of the Istituto Superiore di Sanità - Italian National Institute of Health (contact via email: segreteria.comitatoetico@iss.it) for researchers who meet the criteria for access to confidential data.

We have now included a brief paragraph in the Discussion section detailing how the findings reported in the current manuscript differentiate from and enhance those published by the same working group regarding peripartum hysterectomy due to obstetric hemorrhage within seven days from delivery in Italy (Maraschini et al., Acta obstetricia et gynecologica Scandinavica. 2020).

Journal requirements

1. We ensure that our manuscript meets PLOS ONE’s style requirements.
2. The reference list has been reviewed and we ensure it is complete and correct. None of the cited papers has been retracted.
3. Women included in the study sought treatment in the participating centers between September 1^st^, 2014 and August 31^st^, 2016. Data were fully anonymized before being accessed and analyzed. Since data were analyzed anonymously, need for informed consent was waived by the Ethics Committee of the Istituto Superiore di Sanità - Italian National Institute of Health.
4. All the individual authors of the Regional Obstetric Surveillance System Working Group have now been added, alongside their affiliations, to the Acknowledgments section of our manuscript. The lead author for this group is the senior author of the manuscript, Dr. Serena Donati. We have now specified this information and added her contact email address.

----------------------------------

Dear Referees:

Thank you for the strong positive comments on the first submission of our manuscript. We also appreciate the helpful suggestions as to how we might further improve the paper. Below we respond to each suggestion with details about how we amended the manuscript.

**Response to referees.**

**Response to referee 1.**

We appreciate the comment from referee 1 congratulating us on a well written manuscript.

The referee pointed out the high number (61/384, 15.9%) of missing values for the variable ‘blood loss at delivery’ and how this should be highlighted in the Strengths and Limitations section of our manuscript.

Considering the potential for bias due to >10% missingness, we have now included this as a limitation of our research work. However, we also highlighted that the lack of missingness in data regarding first- and second line treatments of PPH has possibly limited the chance of a biased result interpretation.

Referee 1 asked about the role of placenta previa or low-lying as risk factor for PAS since Table 1 shows a relative risk of 227 associated with not having placenta previa or low-lying.

We apologize for the mistake; the relative risk of 227 is associated with having the condition. This has now been corrected in Table 1.

The referee observed that the number of manual removals in the non-suspected group is much higher than in the group with PAS suspicion and this could be the clinical manifestation of another degree of PAS.

We agree with the referee’s observation. Yet, we would like to point out that unsuspected women were also more likely to receive second-line uterotonic drugs, whereas women with antenatal suspicion were more frequently managed with vascular embolization and hysterectomy (Table 2). These data suggest that in the clinical context of a retained placenta, with or without hemorrhage, clinicians might be more prone to manage the condition as subsequent to uterine atony (the most frequent cause) if no antenatal suspicion of PAS is present. Of note, thirty out of 32 unsuspected women with manual removal of the placenta in our cohort delivered vaginally. In addition, when assessing histologically confirmed PAS cases, we did not identify any difference regarding the grade of invasion between women with and without antenatal suspicion, although we are aware that lack of histological report in some of the cases might have affected these findings.

Referee 1 mentioned that alternative ways to hysterectomy for PAS management, including focal resection and leaving the placenta *in situ* (for cases of placenta percreta), exist but these were not reported in our manuscript.

In our cohort there were four women managed conservatively: three had a partial placenta accreta diagnosed after delivery and only the abnormally adherent cotyledon was left *in situ* whereas the remaining one had an antenatal diagnosis of complete placenta previa with signs of percretion and no attempt of placenta removal was performed at the time of cesarean section. There were no women in whom the focal resection technique was applied.

We have now included these data in the Results section.

**Response to referee 2.**

We thank the referee 2 for highlighting that this is a large-scale, population-based study, which can provide useful information to guide national initiatives.

Referee 2 asked whether patients with (1) severe postpartum hemorrhage, defined as “hemorrhage within 7 days from delivery requiring ≥4 units of whole blood or packed red blood cells”; (2) “hemorrhage due to complete or incomplete uterine rupture”; (3) “peripartum hysterectomy within 7 days from delivery”; and (4) PAS, clinically defined as “difficult or incomplete manual removal of the placenta following vaginal delivery and the need of blood transfusion within 48 hours” or “difficult removal of the placenta during cesarean delivery and clinical evidence of an abnormally invasive placenta”, were all included in the present study.

If not, the referee was wondering how PAS was diagnosed in patients with (1), (2), and (3).

PAS was diagnosed clinically, as defined in (4). All women reported as having PAS by the reference clinician in the participating centers were included in the present study, independent of the associated outcomes, such as severe postpartum hemorrhage (1), uterine rupture (2), and peripartum hysterectomy within 7 days from delivery (3), leading to inclusion in the wider ItOSS research project on severe maternal morbidity due to obstetric hemorrhage.

This has been now clarified in the text.

The referee suggested we provide a better description of Figure 1 in the text and we rearrange it in a more understandable way.

We thank the Referee for this comment. We have now included a brief description of Figure 1 in the Results section and amended both the Figure and its caption to improve clarity.

Referee 2 pointed out that Table 1 reports a 35.4% rate of delivery in Southern Italy.

This was a mistake: rate of delivery in Southern Italy was 64.6% whereas rate of delivery in Northern Italy was 35.4%. This has now been corrected in Table 1.

The referee was wondering whether the reported rate of antenatal suspicion (50%) might be too low, considering the current ultrasound techniques that allow an in-depth investigation of the placenta during gestation.

We agree with the referee that a detailed ultrasound scan, particularly in high-risk women, is pivotal in antenatally suspecting PAS through identification of suggestive signs, such as the loss of the clear zone, the presence of placental lacunae, and the interruption of the bladder-uterus border at grey-scale investigation, and the increased vascularity in the area at Doppler assessment. We also recognize that these ultrasound signs are included in several guidelines on the topic.

However, we kindly disagree with the referee suggesting that a 50% rate of antenatal diagnosis is too low. This rate is similar to that reported by the UKOSS study (66/134, 49.3%) (Fitzpatrick et al., 2014) and the French study (106/242, 43.8%), (Kayem et al., 2021) and substantially higher than that of the NOSS study (60/205, 29.3%) (Thurn et al., 2016), which all had a time period for women’s inclusion in the project similar to ours.

It is likely that currently ongoing research works on PAS, including women in more recent years, might show higher rates of antenatal suspicion, given the increased prevalence and thus awareness of this condition and the institution of dedicated PAS diagnostic services for high-risk women in tertiary care centers, as recently reported by Bhide and colleagues. (Coutinho et al., 2021).

Referee 2 suggested to modify the title of Table 3 to improve clarity.

We appreciate the referee’s suggestion and we have now changed the title as per the referee’s indications.

The referee pointed out the presence of expression disorders and grammatical errors in the manuscript.

The manuscript has now been proofread by a native English speaker, thus hopefully improving its readability.

**References (almost all of these citations are also included in the manuscript)**

Coutinho CM, Giorgione V, Noel L, Liu B, Chandraharan E, Pryce J, Frick AP, Thilaganathan B, Bhide A (2021) Effectiveness of contingent screening for placenta accreta spectrum disorders based on persistent low-lying placenta and previous uterine surgery. Ultrasound Obstet Gynecol 57:91-96.

Fitzpatrick KE, Sellers S, Spark P, Kurinczuk JJ, Brocklehurst P, Knight M (2014) The management and outcomes of placenta accreta, increta, and percreta in the UK: a population-based descriptive study. Bjog 121:62-70; discussion 70-61.

Kayem G et al. (2021) Clinical profiles of placenta accreta spectrum: the PACCRETA population-based study. Bjog.

Thurn L, Lindqvist PG, Jakobsson M, Colmorn LB, Klungsoyr K, Bjarnadóttir RI, Tapper AM, Børdahl PE, Gottvall K, Petersen KB, Krebs L, Gissler M, Langhoff-Roos J, Källen K (2016) Abnormally invasive placenta-prevalence, risk factors and antenatal suspicion: results from a large population-based pregnancy cohort study in the Nordic countries. Bjog 123:1348-1355.
